# Efficacy of ketogenic diet on body composition during resistance training in trained men: a randomized controlled trial

**DOI:** 10.1186/s12970-018-0236-9

**Published:** 2018-07-09

**Authors:** Salvador Vargas, Ramón Romance, Jorge L. Petro, Diego A. Bonilla, Ismael Galancho, Sergio Espinar, Richard B. Kreider, Javier Benítez-Porres

**Affiliations:** 1 EADE-University of Wales Trinity Saint David, Málaga, Spain; 2 Human Kinetics and Body Composition Laboratory, Faculty of Education Sciences, University of Málaga, Málaga, Spain; 3 Research Group in Physical Activity, Sports and Health Sciences, Universidad de Córdoba, Montería, Colombia; 4 Department of Biochemistry and Molecular Biology, Universidad Distrital Francisco José de Caldas, Bogotá, Colombia; 5 BetterbyScience, Málaga, Spain; 6 Exercise & Sport Nutrition Lab, Human Clinical Research Facility, Texas A&M University, College Station, TX USA

**Keywords:** Hypertrophy, Ketosis, High-fat diet, Fat distribution, Bodybuilding

## Abstract

**Background:**

Ketogenic diets (KD) have become a popular method of promoting weight loss. More recently, some have recommended that athletes adhere to ketogenic diets in order to optimize changes in body composition during training. This study evaluated the efficacy of an 8-week ketogenic diet (KD) during energy surplus and resistance training (RT) protocol on body composition in trained men.

**Methods:**

Twenty-four healthy men (age 30 ± 4.7 years; weight 76.7 ± 8.2 kg; height 174.3 ± 19.7 cm) performed an 8-week RT program. Participants were randomly assigned to a KD group (*n* = 9), non-KD group (*n* = 10, NKD), and control group (*n* = 5, CG) in hyperenergetic condition. Body composition changes were measured by dual energy X-ray absorptiometry (DXA). Compliance with the ketosis state was monitored by measuring urinary ketones weekly. Data were analyzed using a univariate, multivariate and repeated measures general linear model (GLM) statistics.

**Results:**

There was a significant reduction in fat mass (mean change, 95% CI; *p*-value; Cohen’s d effect size [ES]; − 0.8 [− 1.6, − 0.1] kg; *p* < 0.05; ES = − 0.46) and visceral adipose tissue (− 96.5 [− 159.0, − 34.0] g; *p* < 0.05; ES = − 0.84), while no significant changes were observed in the NKD and CG in fat mass (− 0,5 [− 1.2, 0.3] kg; *p* > 0.05; ES = − 0.17 and − 0,5 [− 2.4, 1.3] kg; *p* > 0.05; ES = − 0.12, respectively) or visceral adipose tissue (− 33.8 [− 90.4, 22.8]; *p* > 0.5; ES = − 0.17 and 1.7 [− 133.3, 136.7]; *p* > 0.05; ES = 0.01, respectively). No significant increases were observed in total body weight (− 0.9 [− 2.3, 0.6]; *p* > 0.05; ES = [− 0.18]) and muscle mass (− 0.1 [− 1.1,1.0]; *p* > 0,05; ES = − 0.04) in the KD group, but the NKD group showed increases in these parameters (0.9 [0.3, 1.5] kg; *p* < 0.05; ES = 0.18 and (1.3[0.5, 2.2] kg; *p* < 0,05; ES = 0.31, respectively). There were no changes neither in total body weight nor lean body mass (0.3 [− 1.2, 1.9]; *p* > 0.05; ES = 0.05 and 0.8 [− 0.4, 2.1]; *p* > 0.05; ES = 0.26, respectively) in the CG.

**Conclusion:**

Our results suggest that a KD might be an alternative dietary approach to decrease fat mass and visceral adipose tissue without decreasing lean body mass; however, it might not be useful to increase muscle mass during positive energy balance in men undergoing RT for 8 weeks.

## Background

Macronutrient manipulation has become a key nutrition component that, implemented in synergy with training, seeks to improve physical appearance, performance and human health. Among many dietary strategies that have been adopted, ketogenic diet (KD) is a subtype of low-carbohydrate and high-fat diet that needs to be planned considering special dietary features (such as the proportion of macronutrients) and physiological changes (ketosis generation). In view of the foregoing, KD should be planned from an objective perspective, checking for any increase in circulating ketone bodies (KB), a distinctive marker of physiological/nutritional ketosis. Main KB (acetate, acetone, and β-hydroxybutyrate) are produced in the liver under low-carbohydrate availability conditions, acting as an alternative energy source for peripheral tissue, such as skeletal muscle, brain and heart [1]. To achieve a state of ketosis through a KD, carbohydrate intake should be reduced to a maximum of around 50 g per day, or 10% of total caloric intake during the day, while protein intake is moderate or high (e.g. ≈1.2 to 1.5 g∙kg^− 1^⋅d^− 1^). Remaining energy intake is predominantly from fats (≈60 to 80%), depending on the degree of displacement of carbohydrates and proteins [2].

Under normal conditions (with no KD diet or long fasting periods), the circulating KB values (β-hydroxybutyrate being the primary KB) are very low (<3 mmol∙L^− 1^); however, during physiological ketosis, as a result of the KD, ketonemia can reach maximum levels of ≈7–8 mmol∙L^− 1^ with no significant changes in blood pH [3]. At this point, it is important to clarify the difference between physiological ketosis and diabetic ketoacidosis, where the concentration of KB in the blood can exceed ≈20 mmol∙L^− 1^, with a significant reduction in blood pH. In healthy population, the circulating KB values do not exceed ≈8 mmol∙L^− 1^, because the central nervous system uses these molecules efficiently as a source of energy, instead of glucose [4].

Several studies have focused on the effects of KDs on reducing body mass [5, 6], on improving health conditions, or as part of managing certain pathologies such as type 2 diabetes mellitus [7, 8], nervous system disorders such as epilepsy [9–11], and in different types/stages of cancer [12–16]. Currently, there is some controversy surrounding the advantages or disadvantages of KD for sports performance. It has been argued, on the one hand, that there are beneficial effects associated with the reduction of total body mass and body fat, a higher rate of fat oxidation, lower glucose oxidation and a reduction in the rate of muscle glycogen utilization during physical exertion, which represents an advantage in resistance exercise [17]. On the other hand, physiological mechanisms have been cited that may limit performance in resistance training due to central fatigue, possibly because of increased circulation of non-esterified fatty acids which increases competition between these and tryptophan for albumin, resulting in an increase in free tryptophan, which in turn causes a greater absorption by the brain and subsequent augmentation of 5-hydroxytryptamine (serotonin) synthesis, a neurotransmitter linked to the feeling of lethargy and tiredness that may contribute to nerve signal losses at central level and a decrease in motivation. In addition, greater oxidation of amino acids can occur, which increases the concentration of ammonia, contributing to central fatigue [17]. In general, several authors have also established that low-carbohydrate diets or KD do not seem to be superior or offer advantages for resistance exercise, compared with carbohydrate-rich diets [18, 19].

With regards to the effects of KD combined with resistance training (RT), such as muscle hypertrophy, there is even less information available, when compared with studies conducted on endurance-type performance. Even though KD can provide adequate quantities of proteins and calories necessary for muscle-protein synthesis induced by RT, they induce a state similar to fasting, prompting alterations in the metabolic pathways and molecular processes relating to autophagy and stress resistance [20], which consequently might hinder the building of muscle mass.

Considering the need to study on the effects of KD in resistance-trained subjects, the purpose of this study was to determine if following a KD hypercaloric diet would promote greater gains in fat free mass and fat loss during a hypertrophic training period in resistance-trained men. We hypothesized that a KD with caloric surplus in combination with RT in trained men would have a positive impact in fat reduction, and it would benefit the gains in lean body mass (LBM).

## Methods

### Study design

This study was conducted as a randomized, parallel arm, controlled, prospective study. The independent variable was nutritional intervention. The primary outcome variables were changes in body composition.

### Participants

**Fig. 1:**
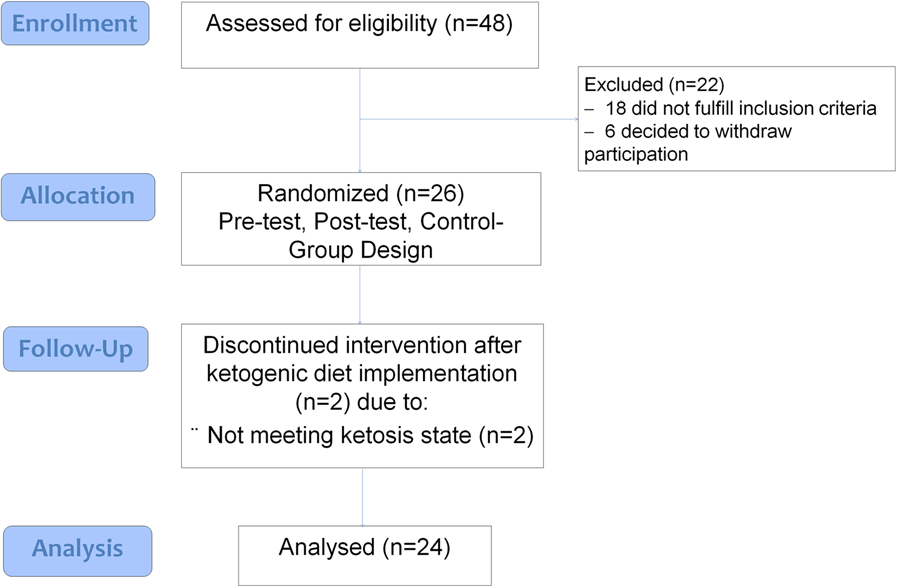
CONSORT diagram

Figure 1 presents a CONSORT diagram. Twenty-four healthy men with more than 2 years of continuous experience in overload training participated in this randomized controlled study (age = 30 ± 4.5 years; height = 177 ± 3.4 cm; weight = 76.7 ± 5.7 kg; BMI = 23.4 ± 2.2 kg/m^2^). All volunteered their participation and agreed to complete the supervised training and diet protocols during the 8 weeks of the study. Subjects who had consumed androgenic-anabolic steroids during the last 2 years or those who consumed any type of dietary supplement during the study were excluded. The subjects were advised of the potential risks of the experiment and signed an informed consent form. The study was developed following the ethical guidelines of the Declaration of Helsinki [21]. The investigation was developed in Málaga (Spain). The first evaluation took place on February 2017 and the second measurement on April of the same year.

### Procedures

#### Body composition

Total and regional body composition were estimated using a Hologic QDR 4500 dual-energy x-ray absorptiometry (DXA) scanner (*Hologic Inc., Bedford, MA, USA*). Each subject was scanned by a certified technician, and the distinguished bone and soft tissue, edge detection, and regional demarcations were done by computer algorithms with APEX Software 3.0 (*APEX Corporation Software, Pittsburg, PA, USA*). For each scan, subjects wore sport clothes and were asked to remove all materials that could attenuate the X-ray beam, including jewelry items. Calibration of the densitometer was checked daily against standard calibration block supplied by the manufacturer.

Abdominal region was delineated by an upper horizontal border located at half of the distance between acromions and external end of iliac crests, a lower border determined by the external end of iliac crests, and the lateral borders extending to the edge of the abdominal soft tissue. All trunk tissue within this standardized height region was selected for analysis. To determine intertester reliability, two different observers selected the area for each subject manually.

#### Nutrition intervention

The participants were randomly assigned to a KD group (*n* = 9), non-KD (NKD) (*n* = 10) group, and control group (CG) (*n* = 5). Compliance with the ketosis state was monitored by measuring urinary ketones weekly using reagent strips (*Ketostix, Bayer Vital GmbH, Leverkusen, Germany*), from week two to the end of the study in KD group. Under the supervision of a registered dietitian, the subjects were given a detailed questionnaire about their work and sociocultural activities, as well as dietary preferences in order to estimate the basal metabolic rate and physical activity-related energy expenditure. Subjects were classified as active in their day-to-day lives, estimating total energy expenditure in line with the indications [22]. Once energy expenditure was determined, together with their weekly training load, a moderate energy surplus was established for experimental groups, since it has been noted that trained men do not require energy increases as high as novice subjects [23, 24]. To guarantee a hyperenergetic condition, a daily energy intake of ≈39 kcal·kg^− 1^·d^− 1^ was used in all subjects. To ensure a maximal anabolic response, NKD group was given a protein intake of 2 g⋅kg^− 1^⋅d^− 1^, as it is recommended for building muscle mass in trained subjects [2, 22, 25], while 25% of total energy intake corresponded to fat and the remaining calories were given in carbohydrates. Macronutrient distribution for NKD group was about 55% CHO; 20% PRO and 25% FAT. On the other hand, ≈42 g total carbohydrates per day were administered to KD group to ensure the ketosis state [26, 27]. Protein intake was 2 g⋅kg^− 1^⋅d^− 1^, and the remaining calories were given in fat with a estimating of 3.2 g∙kg^− 1^⋅d^− 1^. Macronutrient distribution for KD group was about <10% CHO; 20% PRO and 70% FAT. Ad libitum meal timing and frequency throughout the day was allowed to improve dietary adherence. Even though a specific number of meals per day is not necessary, provided the daily energy intake is guaranteed [22], from 3 to 6 meals were recommended, with the respective foods selected for the KD group.

#### Training protocol

**Fig. 2:**
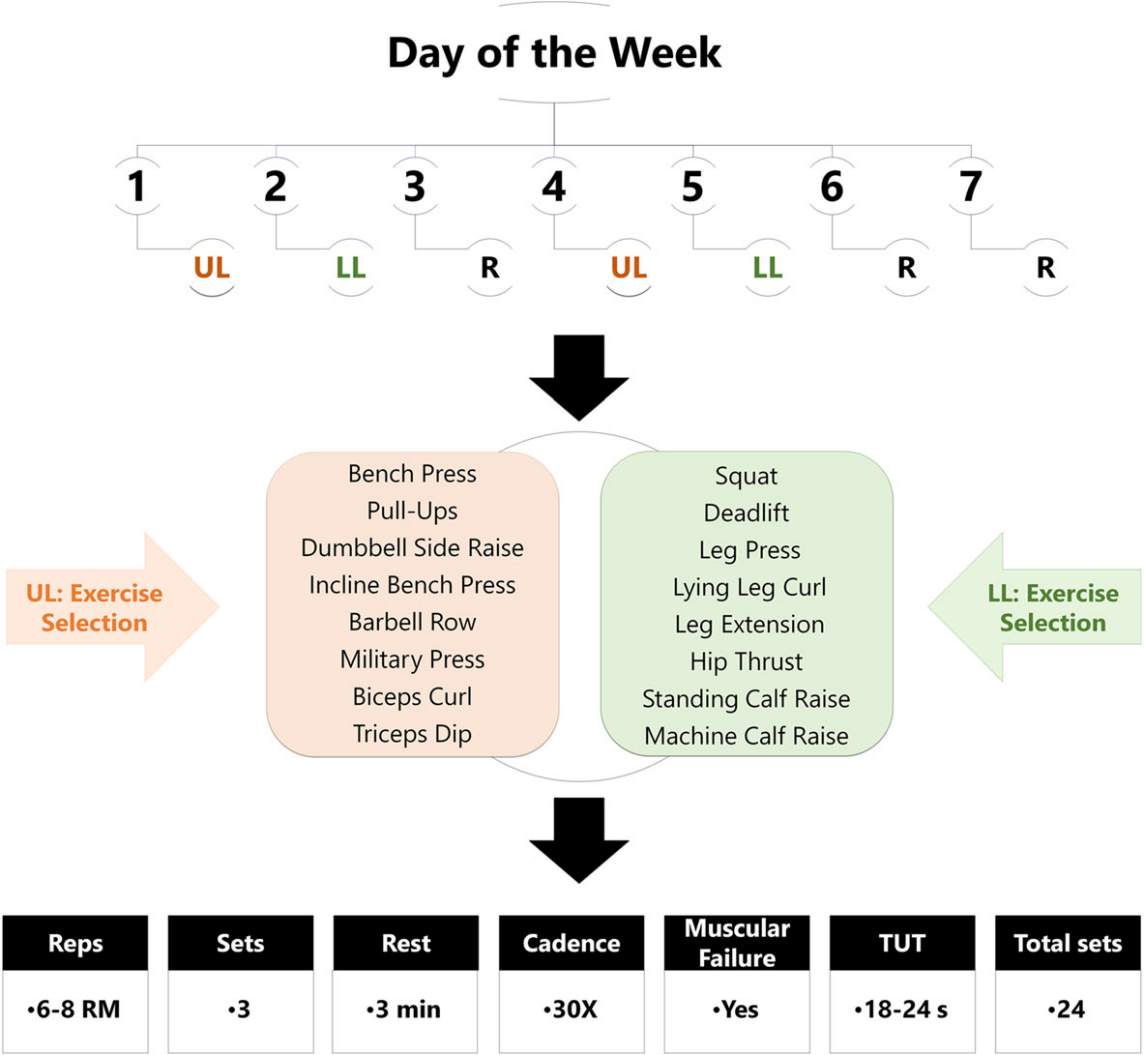
Overview of training protocol. WK: Workout (microcycle); UL: Upper-Limb; LL: Lower-Limb; R: Rest; 30X: 3 s of eccentric contraction and explosive movement during concentric activity

During 8 weeks both KD and NKD groups completed four sessions per week of a hypertrophy training protocol, organized into a 2-days upper- and 2-days lower-limb, with 72 h of rest between sessions to encourage recovery [28] (Fig. 2).

Participants were experienced in overload training and used to different nutritional strategies; therefore, no familiarization session was necessary. Moderate to high loads were used to encourage mechanical tension [29]. Rest between sets lasted 3 min, so that volume did not decline [30, 31]. Cadences were explosive in the concentric activation, and 3 s long during the eccentric contraction to generate more muscle damage [29, 32]. Two weekly stimuli were provided for each muscle group in order to optimize the final results [33]. Push and pull exercises were interspersed for better recovery [34]. Subjects from both groups were asked to increase loads as long as they exceeded repetition rates and had no error technique. During the intervention, all participants were monitored by an RT specialist who supervised and checked the load at each training session, and made the relevant adjustments when was necessary. Meanwhile, men in control group were asked to maintain their current level of physical activity during the study.

#### Statistical analysis

Descriptive statistics tests were applied (mean and standard deviation, SD). Data were analyzed using a univariate, multivariate and repeated measures general linear model (GLM), with two levels by time (pre- and post-test) and considering groups (KD, NKD and CG) as inter-subjects factor. Wilks’ Lambda multivariate tests are reported to describe overall effects of related variables analyzed. Greenhouse-Geisser univariate tests with least significant difference and post-hoc comparisons (Bonferroni correction) are presented for individual variables analyzed. Partial eta squared effect sizes (ηp^2^) were also reported on select variables as an indicator of effect size (ES) of the repeated measures GLM. An Eta squared around 0.02 was considered small, 0.13 medium, and 0.26 large [35]. Furthermore, one-way analysis of variance (ANOVA), with a 95.0% confidence level and Bonferroni post-hoc correction, as is recommended for these studies [36, 37], was performed to detect between-group differences in the Δ changes (post-test – pre-test). In addition, ES calculation was done with Cohen’s *d*, as a standardized measurement based on SD differences; while d = 0.2 was considered a small effect, d = 0.5 was a medium effect and d = 0.8 was a large effect, which is used as a guide for substantive significance. The normal Gaussian distribution of the data was verified by the Shapiro-Wilk test. Mean changes with 95% CI’s completely above or below baseline are considered significant changes from baseline. These statistical analyses were performed with licensed Statistical Package for the Social Sciences (SPSS) software (*SPSS 24.0, SPSS Inc., Chicago, USA*) and GraphPad software (*GraphPad Prism 7.03, California, USA*).

## Results

### Baseline characteristics

A total number of 26 individuals met initial screening criteria and consented to participate in the study (Fig. 1). Two participants did not enter into ketosis state and were excluded from the study, which left nine men for analysis in KD group. Statistical analyses were performed on 24 individuals. Descriptive statistics with baseline characteristics are summarized, by groups in Table 1.

**Table 1:** Characteristics of participants at baseline

	CG	KD	NKD	*p*-value
Age (years)	31.6 ± 4.6	27.6 ± 4.2	27.1 ± 5.6	0.276
Height (cm)	179.9 ± 7.8	178.3 ± 4.0	178.3 ± 6.2	0.873
BW (kg)	78.9 ± 6.5	78.8 ± 7.8	74.6 ± 5.3	0.306
BMI (kg∙m^2^)	24.5 ± 1.7	24.4 ± 2.6	23.9 ± 1.6	0.793
FM (kg)	13.4 ± 4.5	12.0 ± 2.7	11.3 ± 2.6	0.499
LBM (kg)	65.6 ± 2.6	66.8 ± 6.8	63.2 ± 4.4	0.350
VAT (g)	757.7 ± 265.3	688.9 ± 125.4	658.0 ± 200.5	0.650

Data are means ± SD; *p* < 0.05 is considered significant; *BW* Total body weight, *BMI* Body Mass Index, *FM* Fat mass, *LBM* Lean body mass, *VAT* Visceral adipose tissue

### Body composition

The statistical results before and after the intervention for total body weight (BW) and body composition; fat mass (FM), visceral adipose tissue (VAT), and LBM are shown in Table 2. Multivariate analysis showed significant overall Wilks’ Lambda in time interaction (*p* = 0.031; with a large effect size, ηp^2^ = 0.36) and in time x group (*p* < 0.05; with a large effect size, ηp^2^ = 0.264). On the other hand, univariate analysis revealed significant differences in time x group interaction between BW, and LBM (*p* < 0.05), with a large effect size for BW (ηp^2^ > 0.36); however, no significant differences were found in VAT. Significant differences were observed over time in VAT and LBM, with medium effect size for both (ηp^2^ = 0.20 and 0.23, respectively). No significant differences were found after group interaction analysis of the study variables.

**Table 2:** Results before and after the intervention for body composition by groups

		Pre	Post	ES	Interaction	*p*-value (ηp^2^)
		(Mean ± SD)	(Mean ± SD)			
BW	CG	78.9 ± 6.5	79.2 ± 6.6	0.05	Time	0.830 (0.002)
	KD	78.8 ± 7.8	77.4 ± 7.9	−0.18	Group	0.437 (0.076)
	NDK	74.6 ± 5.3	75.5 ± 4.9*	0.18	Time x Group	0.016 (0.327)
FM	CG	13.4 ± 4.5	12.8 ± 4.0	−0.12	Time	0.013 (0.258)
	KD	12.0 ± 2.7	10.9 ± 2.2*	−0.46	Group	0.457 (0.072)
	NDK	11.3 ± 2.6	10.9 ± 2.7	−0.17	Time x Group	0.447 (0.074)
VAT	CG	757.7 ± 265.3	759.4 ± 317.2	0.01	Time	0.031 (0.203)
	KD	688.9 ± 125.4	592.4 ± 103.1*	−0.84	Group	0.490 (0.066)
	NDK	658.0 ± 200.5	624.2 ± 201.5	−0.17	Time x Group	0.130 (0.177)
LBM	CG	65.6 ± 2.6	66.4 ± 3.5	0.26	Time	0.023 (0.224)
	KD	66.8 ± 6.8	66.5 ± 6.9	−0.04	Group	0.516 (0.061)
	NDK	63.2 ± 4.4	64.6 ± 4.2*	0.31	Time x Group	0.025 (0.297)

Data are means ± SD; Greenhouse-Geisser univariate *p*-levels are presented for each variable; *p* < 0.05 is considered significant; (*) denotes a significant difference from baseline; *ES* Effect Size (Cohen’s d), *BW* Total body weight, *FM* Fat mass, *VAT* Visceral adipose tissue, *LBM* lean body mass

According to the results by group, BW increased in KD group (*p* < 0.05), but to a small size (ES = 0.18), with no significant differences in the other groups (NKD and CG). With regards to FM, only KD group showed a significant reduction (*p* < 0.05), expressing a medium effect (ES = − 0.46). Similarly, VAT only decreased markedly in the KD group (*p* < 0.05), showing a considered large effect (ES = − 0.84). Conversely, LBM showed a highly significant increase (*p* < 0.05) with moderate effect (ES = 0.31) in the NKD group; however, although LBM decreased in the KD group, this did not represent a statistically significant difference or significant effect (*p* > 0.05; ES = − 0.04).

**Fig. 3:**
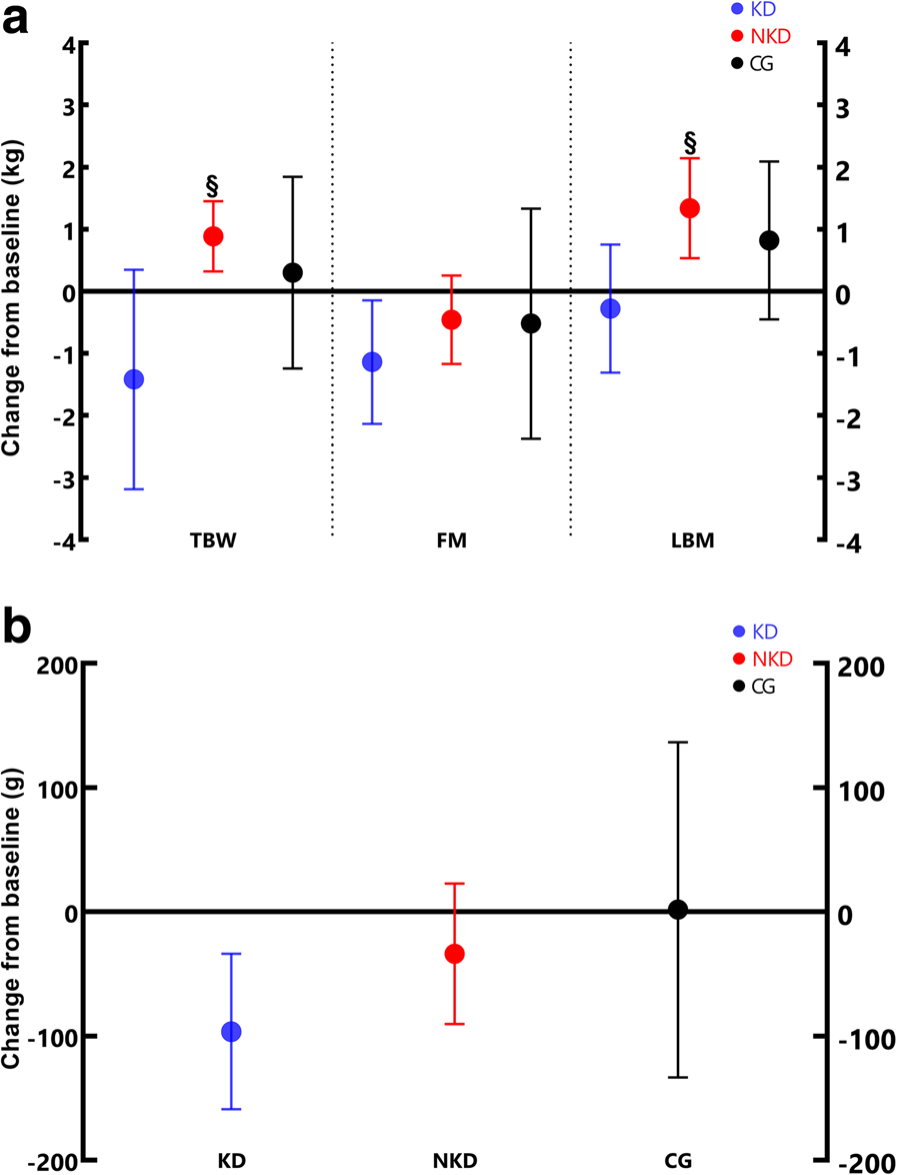
Changes in body mass and body composition. Mean changes with 95% CI’s completely above or below the baseline are significant changes; BW: Total body weight; FM: Fat mass; VAT: Visceral adipose tissue; LBM: lean body mass. **a** Changes in BW, FM, LBM. **b** Changes in VAT. ǂ Significant difference with KD after post-hoc analysis (*p* < 0.05)

These results suggest that KD group achieved a positive change in body composition, due to a decrease in BW (− 0.9 [− 2.3, 0.6] kg; *p* > 0.05) with a reduction in FM (− 0.8 [− 1.6, − 0.1] kg; *p* < 0.05) and accompanied by a notably lower VAT (− 96.5 [− 159.0, − 34.0] g; *p* < 0.05). Regarding to LBM, an adequate carbohydrate intake (non-ketogenic or conventional dietary approach), in conjunction with a caloric surplus and a higher protein intake, might be the most viable option for inducing muscle hypertrophy after RT. This last was shown in this study, where there was an increase in LBM (1.3 [0.5, 2.2] kg; *p* < 0.05) in the NKD group, leading to an increase in BW (− 0.9 [− 2.3, 0.6] kg; *p* < 0.05). Figure 3 shows significant differences in BW and LBM for NKD group; and FM and VAT for KD group. Likewise, post-hoc analysis showed significant difference in the BW and LBM between KD and NDK groups.

## Discussion

The aim of this study was to determine the efficacy of the KD when combined with an RT program on body composition in trained subjects over a period of 8 weeks of intervention.

We originally hypothesized that this intervention would improve body composition due to a greater reduction in FM and VAT, and an increase in LBM. Our hypothesis is supported by some lines of evidence, but there are contradictory findings due to a lack of studies analyzing the effects of the KD (with and without RT protocol) on FM, VAT and muscle hypertrophy. Human studies have reported a reduction in FM during and after KD, but with a concomitant loss of of LBM [38–44]. For example, Gomez-Arbelaez [45], found that a low-calorie KD (starting in the initial phases with ≈600–800 kcal per day and following the PNK® method) resulted in a decrease in VAT, according to a follow-up study performed over 4 months. Notwithstanding, it should be noted that these studies included obese subjects, in some cases with at least one cardiovascular risk factor and, with no physical exercise intervention, strength training in particular. In another study [46], there was a reduction in adipose mass tissue and a parallel increase in LBM after performing a variety of strength or resistance exercises in moderately active subjects with normal weight; these changes in body composition (especially FM reduction) were attributed in part to a decrease in insulin concentrations. It is probable that the incorporation of RT, together with moderate/high protein consumption and a caloric surplus, may be an important strategy for maintaining fat free mass during KD. In particular, RT alone, or combined with endurance training, accompanied by a hypoenergetic KD, might be useful for the preservation of fat free mass and the increased metabolic rate in obese subjects, as an intervention that deserves further research, considering the complexity of this multifactorial illness [47]. In fact, even though endurance exercise is more effective than RT in reducing VAT [48], a combination of endurance training and RT is more plausible for improving body composition in this population [49]. Since few studies have evaluated the combined effect of the KD and RT in trained subjects on VAT, our study contributes to current literature by showing a significant reduction in VAT after 8 weeks of KD in hyperenergetic condition in resistance-trained men. These results suggest that KD group achieved a positive change in body composition, due to a decrease in BW (− 0.9 [− 2.3, 0.6] kg; *p* > 0.05) with a reduction in FM (− 0.8 [− 1.6, − 0.1] kg; *p* < 0.05) and accompanied by a notably lower VAT (− 96.5 [− 159.0, − 34.0] g; *p* < 0.05). This supports the need for in-depth analysis about the importance of macronutrient distribution, comparing isoenergetic nutritional programs, on the distribution of body fat.

On the other hand, animal studies on ketosis-induced interventions after KD have not found neither acute nor chronic changes in hypertrophic response in skeletal muscle, when strength exercises were performed, in comparison with a mixed diet of macronutrients [50]; however, a reduction in FM was observed in these rodents [51]. Although these results were obtained in animal models, it seems that these effects are similar but not extrapolable to humans. Our study involved resistance-trained young men with an RT program intervention focused on mechanical tension to generate changes in LBM, considering this as one of the main factors of RT-induced muscle hypertrophy [29, 52, 53]. Also, a 3 min-rest pause between sets and short time under tension was considered, to discourage a dramatic decrease in muscle glycogen. Subsequently, comparison of changes in variables, by one-factor ANOVA, revealed a difference between means in all groups regarding BW and LBM; in fact, there was an increase in LBM (1.3 [0.5,2.2] kg; *p* < 0.05) in the NKD group, leading to an increase in BW (− 0.9 [− 2.3, 0.6] kg; *p* < 0.05). Figure 3 shows significant differences in BW and LBM for NKD group; and FM and VAT for KD group. Likewise, post-hoc analysis showed significant difference in the BW and LBM between KD and NDK groups. These results are in agreement with those obtained by Rauch et al. [54], who compared the effects of a KD (5% CHO, 75% fat and 20% protein) with a traditional western diet (55% CHO, 25% fat and 20% protein) in men undergoing RT training (*n* = 26), during 11 weeks. These authors also found a decrease in FM in the KD group but, unlike our results, there was an increase in LBM.

The results of the present study are in accordance with the preliminary hypothesis, since analysis of the data showed a significant reduction in FM and VAT in resistance-trained men undergoing a KD while participating in a RT program; however, no changes were seen in LBM in this group. The clinical significance is the reduction in VAT, which could have health benefit because of its inverse correlation to cardiometabolic disease [55, 56]. Regarding to LBM, an adequate carbohydrate intake (non-ketogenic or conventional dietary approach), in conjunction with a caloric surplus and a higher protein intake, might be the most viable option for inducing muscle hypertrophy after RT.

## Limitations

This study has several limitations that should be mentioned. Firstly, this research only included body composition measurements and did not include blood measures. In addition, limited outcome measurements, small number of subjects and intervention time (8 weeks) reduce the impact of the study. On the other hand, dietary assessment of appetite suppression by high-fat diet was not performed. So, it is possible to have variations in energy intake even though participants were instructed to follow specific dietary recommendations. Moreover, since KD may affect negatively training volume, we should consider integrating performance measurements or load volume to see changes. In addition, rated perceived exertion might give interesting information about changes during KD adaptation and progression of RT protocol.

## Conclusions

According to our results, we concluded that subjects who underwent RT during a KD experienced a greater reduction in FM and VAT, when compared to the NKD group. The greater reduction in VAT may have some clinical relevance due to its inverse association to cardio-metabolic risk. Further studies are necessary to evaluate the advantages of this combination (RT and KD) in subjects with excess of body FM, with particular attention to the reported significant reduction in VAT, which might be highly beneficial to this population given that LBM is maintained. Indeed, this research showed no significant changes nor effect size on LBM, despite hyperenergetic condition and high protein intake (2.0 g∙kg^− 1^⋅d^− 1^) in resistance-trained men of the KD group. Thus, we conclude that low-carbohydrate dietary approaches, such as KD, would not be an optimum strategy for building muscle mass in trained men under the training conditions of this study (mechanical tension-focused RT protocol during 8 weeks).

## Abbreviations

ANOVA: One-way analysis of variance
BMI: Body mass index
BW: Body weight
CG: Control group
DXA: Dual-energy x-ray absorptiometry
ES: Effect size
FM: Fat mass
KB: Ketogenic bodies
KD: Ketogenic diet
LBM: Lean body mass
NKD: Non-ketogenic diet
RT: Resistance training
VAT: Visceral adipose tissue
ηp^2^: Partial eta squared effect size
